# Schroth Physiotherapeutic Scoliosis-Specific Exercises Added to the Standard of Care Lead to Better Cobb Angle Outcomes in Adolescents with Idiopathic Scoliosis – an Assessor and Statistician Blinded Randomized Controlled Trial

**DOI:** 10.1371/journal.pone.0168746

**Published:** 2016-12-29

**Authors:** Sanja Schreiber, Eric C. Parent, Elham Khodayari Moez, Douglas M. Hedden, Douglas L. Hill, Marc Moreau, Edmond Lou, Elise M. Watkins, Sarah C. Southon

**Affiliations:** 1 Faculty of Rehabilitation Medicine, University of Alberta, Edmonton, Alberta, Canada; 2 Department of Physical Therapy, University of Alberta, Edmonton, Alberta, Canada; 3 School of Public Health, University of Alberta, Edmonton, Alberta, Canada; 4 Department of Surgery, University of Alberta, Alberta Health Services, Edmonton, Alberta, Canada; 5 Alberta Health Services, Edmonton, Alberta, Canada; 6 Glenrose Rehabilitation Research Centre, Alberta Health Services, Edmonton, Alberta, Canada; Bern University of Applied Science, SWITZERLAND

## Abstract

**Background:**

The North American non-surgical standard of care for adolescent idiopathic scoliosis (AIS) includes observation and bracing, but not exercises. Schroth physiotherapeutic scoliosis-specific exercises (PSSE) showed promise in several studies of suboptimal methodology. The Scoliosis Research Society calls for rigorous studies supporting the role of exercises before including it as a treatment recommendation for scoliosis.

**Objectives:**

To determine the effect of a six-month Schroth PSSE intervention added to standard of care (Experimental group) on the Cobb angle compared to standard of care alone (Control group) in patients with AIS.

**Methods:**

Fifty patients with AIS aged 10–18 years, with curves of 10°-45° and Risser grade 0–5 were recruited from a single pediatric scoliosis clinic and randomized to the Experimental or Control group. Outcomes included the change in the Cobb angles of the Largest Curve and Sum of Curves from baseline to six months. The intervention consisted of a 30–45 minute daily home program and weekly supervised sessions. Intention-to-treat and per protocol linear mixed effects model analyses are reported.

**Results:**

In the intention-to-treat analysis, after six months, the Schroth group had significantly smaller Largest Curve than controls (-3.5°, 95% CI -1.1° to -5.9°, *p* = 0.006). Likewise, the between-group difference in the square root of the Sum of Curves was -0.40°, (95% CI -0.03° to -0.8°, *p* = 0.046), suggesting that an average patient with 51.2° at baseline, will have a 49.3° Sum of Curves at six months in the Schroth group, and 55.1° in the control group with the difference between groups increasing with severity. Per protocol analyses produced similar, but larger differences: Largest Curve = -4.1° (95% CI -1.7° to -6.5°, *p* = 0.002) and $\sqrt{\text{Sum of Curves}}=-0.5{}^{\circ}$ (95% CI -0.8 to 0.2, *p* = 0.006).

**Conclusion:**

Schroth PSSE added to the standard of care were superior compared to standard of care alone for reducing the curve severity in patients with AIS.

**Trial Registration:**

NCT01610908

## Introduction

Adolescent idiopathic scoliosis (AIS), a three-dimensional torsional deformity of the spine and trunk[1], is the most common (84%-89%) form of scoliosis[2] with a prevalence between 0.47 and 5.2% in the general adolescent population.[3] There is a high predominance of AIS among girls, rising with higher severity of the curve.[3] The risk of progression is linked to the remaining growth potential and initial curve magnitude.[4] Scoliosis may lead to mental health concerns,[5] pain,[6,7] respiratory complications,[8] and limited function.[6,7] The negative consequences usually manifest once the curve exceeds 30°[9]. It is generally agreed that curves less than 30° are unlikely to progress after skeletal maturity.[10] Therefore, early treatment is recommended throughout pubertal growth to prevent progression.

In North America, the Scoliosis Research Society (SRS) developed standard of care guidelines for growing patients with AIS, which includes observation (curves 10° to 25°), bracing (curves 25° to 45°), and elective surgery (curves >45°).[11] Some scoliosis centers are more proactive, starting bracing with curves under 25° that have demonstrated progression.[12]

The efficacy of exercise treatment is controversial. Although evidence suggests that PSSE, which include auto-correction in 3D, integration in daily life, stabilizing the corrected posture, and patient education,[1,13] could improve some outcomes,[14] PSSE have not yet been widely accepted in North America. However, the international Society on Scoliosis Orthopaedic and Rehabilitation Treatment (SOSORT) that has interest in non-operative management of patients with scoliosis, developed guidelines[1,15,16] that recommend PSSE used alone and as an add-on to bracing for patients with curves <45° to 1) prevent further curve progression at puberty, 2) to prevent or treat respiratory dysfunction, 3) to prevent or treat spinal pain syndromes, 4) to improve aesthetics via postural correction, and 5) reduce the need for surgery. [1,13] Differences between the North American and European guidelines may be due to cost, culture, social standards or, possibly differing appraisals of the quality of research involving exercises.

Bracing can induce stress, fear of injury, discomfort, limitation in activities, negative self-esteem[17] and impair lung function,[18]. While surgery reduces deformity, it does not necessarily improve other outcomes.[19] Moreover, patients fear surgery due to its invasiveness, risk of complications, post-surgical pain, and long recovery. Conversely, exercises are well received,[20] and frequently requested by patients and their parents.[21]

Several systematic reviews on exercises for scoliosis [14,22–24] report promising results on curve severity, such as improving neuromotor control, respiratory function, back muscle strength, and cosmetic appearance. However, most reviews [14,22–24] carry a risk of reviewer bias because they were published by authors of studies included in the reviews. In a recent independent review,[25] nine prospective cohort studies were included, of which only three were controlled and only one used observer blinding. Other limitations of exercise studies included unclear reporting of patient selection criteria, recommendations for, and contraindications to exercise, not reporting on compliance, intention-to-treat analyses, or recruitment strategies. Change in Cobb angles was usually statistically significant, but often within the measurement error. Most recently an overview of systematic reviews on non-surgical interventions for AIS analyzed 21 reviews and concluded that there is insufficient evidence to support the use of non-surgical treatments, including exercises, for AIS.[26]

Among the promising PSSE approaches reviewed, Schroth exercises were the most studied. The Schroth method consists of sensorimotor, postural and breathing exercises aimed at recalibration of normal postural alignment, static/dynamic postural control, and spinal stability.[27] Several studies of limited quality demonstrated positive outcomes of Schroth exercises on back muscle strength,[28] breathing function,[28] slowing curve progression,[29] improving Cobb angles,[28,29] and decreasing the prevalence of surgery.[30] Recently, a 6-month long randomized controlled trial (RCT) compared the efficacy of a supervised to non-supervised Schroth intervention in patients with AIS, while a control group received no treatment.[31] Of 45 participants with AIS, 15 were randomized into each of the groups. After six months, the supervised Schroth exercises were superior in improving Cobb angles, scoliometer measures, waist asymmetry and rib hump compared to the non-supervised and no-treatment groups. However, the authors did not report on blinding the outcome assessors and did not quantify the compliance.

To strengthen the existing evidence on PSSE, we conducted this RCT to determine the effect of a six-month Schroth PSSE intervention added to standard of care (observation or bracing) on the Cobb angle, compared to the standard of care alone in patients with AIS. We hypothesized that Schroth PSSE would improve scoliosis curves.

## Methods

### Study design

**Fig 1:**
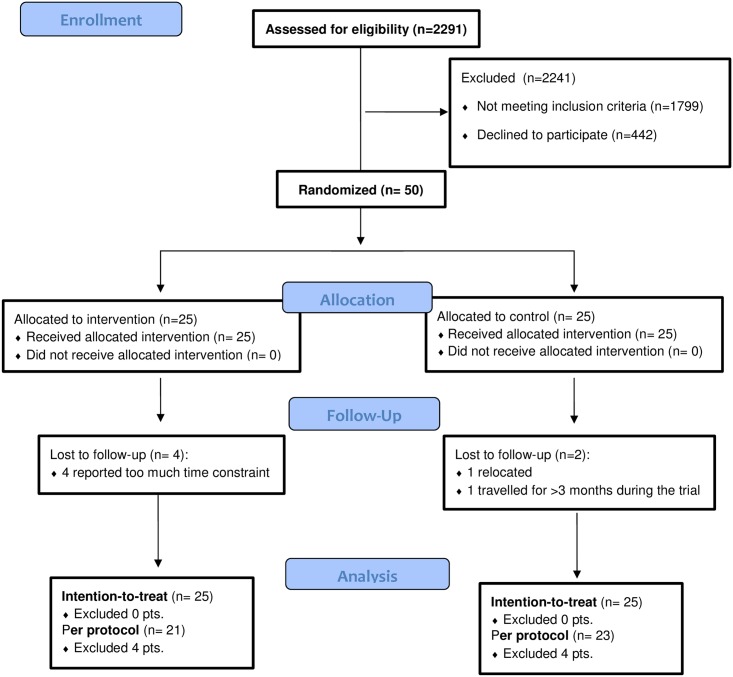
CONSORT flow diagram.

This was a parallel, phase II, assessor and statistician blinded, randomized controlled clinical trial (ratio 1:1). The protocol has been published.[32] The CONSORT flow diagram and checklist are available in Fig 1 and in S1 Fig, respectively.

### Participants and therapists

Between April 2011 and November 2013, 50 patients with AIS were enrolled from a regional University of Alberta Hospital—Scoliosis Clinic in Edmonton, Canada. A primary care practitioner can refer patients to the Scoliosis Clinic if scoliosis is suspected. The patients’ evaluations within the trial occurred between April 2011 and May 2014. The local Health Research Ethics Board Biomedical (HREB) approved the study prior to beginning patient recruitment, on September 16, 2010 (Pro00011552). However, our trial was registered later (April 2012) in the registry of clinical trials (ClinicalTrials.gov, Trial registration: NCT01610908), because when the recruitment started in 2011, we were not aware that the trial needed to be registered beyond being approved by the Health Research Ethics Board. Despite the late registration, our trial was conducted according to the approved Ethics/registered protocol, available in supporting S1 File. In addition, all ongoing and related trials for this intervention are registered (ClinicalTrials.gov, Trial registration: NCT01610908).

The interdisciplinary care team at the clinic consists of pediatric spinal surgeons, nurse practitioners, engineers, and orthotists. A surgeon or a nurse diagnoses and prescribes a scoliosis care plan typically consisting of further investigation (when appropriate), observation, bracing or surgery. The clinic can refer patients for general physiotherapy, or psychology as deemed necessary.

**Inclusion criteria** were: 10–18 years old patients with AIS, both genders, all curve types, curves between 10°-45°, Risser grade 0 to 5, with or without brace, and the ability to attend weekly visits. Risser grades refer to a child's' skeletal maturity, where children with Risser 0 and 1 are growing rapidly and are considered skeletally immature, and patients who are Risser 4 and 5 have stopped growing and are considered skeletally mature.[33] **Exclusion criteria** were: patients with diagnosis other than AIS, having completed brace treatment, scheduled for surgery, a follow-up scheduled later than 6±2 months, and previous spine surgery. We obtained written informed assent from patients and written informed parental consent.

Prior to this study, the primary therapist (SS) had three years of Schroth therapy experience and provided approximately 95% of the therapy sessions. A second certified therapist (ECP) filled-in as needed.

### Randomization and masking

Before the weekly scoliosis clinics, a research coordinator screened all attending patients for eligibility. After being seen by a surgeon or a nurse, patients who previously expressed interest in participation in any type of research conducted at the clinic were approached. The research coordinator explained the study and invited consecutive eligible patients to participate. The research coordinator, the surgeons and the nurse were not involved in the randomization, treatment or outcome assessments. Within two weeks from this visit, a researcher obtained consent and booked an evaluation to confirm eligibility. During the initial visit to our lab (University of Alberta, Rehabilitation Sciences), an independent blinded evaluator completed the baseline exam, and a Schroth therapist who provided treatments determined a scoliosis curve type using our Schroth classification algorithm designed for this study. The participants were then randomized using a computer-generated sequence contained in pre-sealed sequentially numbered opaque envelopes into the Schroth exercises or the control group. Random size (4–8) blocked randomization stratified for the four Schroth curve types was used to ensure a balanced allocation of curve types in both groups (25/group).

Therapists and participants could not be blinded to the treatment. Participants were asked not to reveal their group allocation to ensure evaluator blinding. The statistician was also blinded to coding of group allocation. Radiographs were obtained during routine clinic visits by a trained technician blinded to study participation. An experienced evaluator masked to groupings and timing measured the radiographs.

### Intervention—experimental group

The six-month supervised Schroth PSSE intervention included five one-hour long private sessions delivered during the first two weeks, followed by weekly one-hour long group classes combined with a 30–45 min daily home exercise program. Exercises with the corrective movements required, the targeted curve type, the level of passive support involved, whether static or dynamic, and the dosages recommended, as well as the detailed description of the intervention were published previously. [32] A Schroth curve classification algorithm and algorithms to guide the exercise prescription and progression for each Schroth curve type were developed to standardize treatment and ensure reproducibility and were previously published. [32]

Compliance was monitored using logbooks, and verified daily by a parent and weekly by the therapist. Therapists assessed adequate exercise performance weekly using a checklist. Attendance was calculated as a percentage of prescribed visits, and compliance as a percentage of the prescribed exercise dose completed over six months.

### Intervention—control group

Control subjects received the standard of care including observation or bracing with SRS recommended dosage if the SRS bracing criteria were met, and attended only study assessments.

### Measurements

The outcomes included the change in the Cobb angle of the Largest Curve and in the Sum of Curves measuring ≥10° to ensure capturing changes affecting all curves. To quantify the Cobb angles, standing posterior-anterior radiographs were obtained using a positioning frame at baseline and six months. Cobb angles were measured for each curve using semi-automated software with measurement error ≤2.5°.[34]

The Self-efficacy Questionnaire score, collected at baseline was used as a covariate for the analyses. This validated questionnaire measures self-efficacy for overcoming barriers to physical activity (defined as corrective exercises) using eight items rated from one (Disagree a lot) to five (Agree a lot).[35]

Cobb angle outcomes reported here were measured only at baseline and 6-month follow-up. However, a physical exam including height, weight, trunk rotation using scoliometer, Schroth curve classification, and demographics were collected at baseline, three- and six-month follow-ups. At those three time points, we also measured the following secondary outcomes: vertebral rotation, back muscle endurance, Scoliosis Research Society 22r (SRS-22r), Spinal Appearance Questionnaire (SAQ), global rating of change (at three and six months follow-ups), Self-efficacy scores, numeric pain ratings and diagram, and surface topography measures of posture. The back muscle endurance, SRS-22r and SAQ questionnaires, but not curve angles, have been reported separately in a publication preceding the present one.[36] Other outcomes announced in the protocol will be reported in subsequent publications.

### Statistical analysis

Descriptive statistics were calculated for baseline demographics and radiographs, for the entire sample, and for the patients who dropped out.

To assess differences between groups in changes from baseline to six months while adjusting for important covariates, per protocol and intention-to-treat linear mixed effects models analysis were used. Separate analyses were conducted for each outcome. Covariates considered included age, weight, height, self-efficacy, brace-wear (yes/no), and Schroth scoliosis classification. For covariates selection, a stepwise variable selection method using Akaike information criterion (AIC) was used.[37] Several correlation structures were tested for the models for each outcome. The best fitting correlation structure as determined by the AIC was found to be Autoregression—AR (1). Outcomes were transformed as needed to meet normality assumptions. Statistical analyses were performed using R language and environment for statistical computing.[38] In order to control for the familywise Type I error, we used Holm-Bonferroni sequential correction).[39]

### Sample size calculation

To detect a 0.50 effect size when comparing the change in the primary outcome between two groups with 80% power using a two-tailed 0.05 hypothesis test, and considering a 0.6 correlation between repeated measures in two time points, 50 patients per group were needed.[40] However, the study ended after recruiting 50 participants when funding was received to continue the study as a multicenter RCT with slightly different participants’ criteria (Trial registration NCT01610908).

## Results

Groups did not differ at baseline for age, number of braced patients, height, Cobb angles, Risser sign and Lonstein and Carlson risk of progression[41]. However, controls were 4.4 kg heavier than Schroth participants. Forty-seven girls and three boys were evenly distributed between groups. The mean height, weight and age were 1.60 m (SD = 0.1), 48.2 kg (SD = 8.3), and 13.4 years (SD = 1.6), respectively. The mean Largest Curve was 28.5° (SD = 8.8°) and the mean Sum of Curves was 51.2° (SD = 22.3°) with 65% risk of progression[41](Table 1). Raw mean scores and corresponding measures of variability for each outcome and time point are provided in Table 2.

**Table 1:** Baseline characteristics of the study population.

	Schroth exercises + Standard of care (95% Confidence interval), N = 25	Standard of care (95% Confidence interval), N = 25
**Age (years)**	13.5 (12.7–14.2)	13.3 (12.7–13.9)
**Girls n (%)**	23 (92)	24 (96)
**Braced participants n, (%)**	17 (68)	17 (68)
**Height (m)**	1.60 (1.6–1.6)	1.60 (1.6–1.6)
**Weight (kg)**	45.9 (42.6–49.1)	50.5 (47.1–54.0)
**Largest curve (°)**	29.1 (25.4–32.8)	27.9 (24.3–31.5)
**Sum of curves (°)**	48.1 (39.1–57.2)	54.3 (44.9–63.6)
**Risser sign (0 to 5)**	1.76 (1.10 to 2.45)	1.44 (0.77 to 2.11)
**Lonstein and Carlson Risk of progression**[41] **(%)**	65	65

**Table 2:** Raw mean scores for each outcome at baseline and 6-month follow-up. “0”—Standard of care group; “1”—“Schroth + standard of care group.

Outcome	Group	Number of patients	Mean	Standard Deviation	95% Confidence Interval	Minimum	Maximum
**Largest Cobb at Baseline (°)**	0	25	27.9	8.8	24.3–31.5	11.7	42.0
	1	25	29.1	8.9	25.4–32.8	11.3	44.3
	Total	50	28.5	8.8	26.0–31.0	11.3	44.3
**Sum of Curves at Baseline (°)**	0	25	54.3	22.6	44.9–63.6	11.7	95.1
	1	25	48.2	21.9	39.1–57.2	11.3	86.0
	Total	50	51.2	22.3	44.9–57.5	11.3	95.1
**Largest Cobb at 6-months**	0	20	29.1	8.8	25.0–33.3	12.1	44.7
	1	23	27.7	8.9	23.8–31.5	14.4	43.9
	Total	43	28.4	8.8	25.7–31.0	12.1	44.7
**Sum of Curves at 6-months**	0	20	57.5	24.9	45.8–69.1	15.8	102.4
	1	23	45.7	21.4	36.4–54.9	14.4	80.6
	Total	43	51.2	23.6	43.9–58.4	14.4	102.4

Schroth curve types were as follows: 3c (n = 7) affecting the thoracic spine without pelvis imbalance, 3cp (n = 15) thoracic dominant deformity with imbalanced pelvis observed on the thoracic concave side, 4c (n = 5) with a thoracolumbar/lumbar dominant deformity without pelvis imbalance and 4cp (n = 23) with a thoracolumbar/lumbar dominant deformity with pelvis displaced to the lumbar concave side. Curve types were balanced between groups with no more than one subject difference for each type.

### Dropouts

Attrition was 12% (6/50), with four dropouts in the Schroth and two in the control group. Of these, there were four girls (one control and three in the Schroth group) and two boys (one per group). The Largest Curve (23°, SD = 5.3) and Sum of Curves (38°, SD = 17.5) of patients who dropped out were smaller (less severe) than for the remaining patients. The reasons for dropout are reported in Fig 1.

### Compliance

Patients with complete follow-up attended 85% of prescribed visits and completed 82.5% of the home program. Considering the dropouts and assuming zero compliance after the dropout occurred, 76% of visits were attended and 73% of the prescribed home exercises were completed.

### Intention-to-treat analysis

#### Largest curve

The difference in Largest Curve between groups at six months was -3.5° (95% CI -5.9° to -1.1°, *p* = 0.006) with smaller curves in the Schroth PSSE group. On average, after adjusting for confounders the Largest Curve decreased by 1.2° in the Schroth and increased by 2.3° in the control group over six months.

The covariates selected by the model included height, weight, and curve classification. However, only weight and classifications 3cp and 4cp had significant main effects on the Largest Curve. The significant covariates influenced the outcome as follows: 1) for every 1 kg increase in weight, patients had on average 0.44° larger Largest Curve (95% CI 0.04° to 0.82°, *p* = 0.04); and 2) patients classified as 3cp and 4cp had on average 12.1° (95% CI 5.5° to 18.9°, *p* = 0.001) and 8.3° (95% CI 3.0° to 14.9°, *p* = 0.01) larger Largest Curve than patients classified as 3c, respectively (Table 3). No other covariates among those examined including age, self-efficacy or brace wear had significant main effect.

**Table 3:** Linear mixed effects model coefficients and significance values in the intention-to-treat and the per protocol analyses with 95% confidence intervals;.

	Intention to treat (N = 50)			Per protocol (N = 44)		
	Value	95% Confidence interval	p-value	Value	95% Confidence interval	p-value
***Largest Cobb (°)***						
**Interaction group by time**	- 3.53	-5.94 to -1.12	0.006*	-4.13	-6.51 to -1.74	0.002*
**Group**	6.87	1.38 to 12.36	**0.02**	9.00	3.47 to 14.52	**0.003**
**Time**	2.32	0.56 to 4.08	**0.01**	2.31	0.62 to 4.00	**0.01**
**Height**	- 31.88	-65.28 to 7.86	0.13	-31.88	-70.14 to 6.38	0.11
**Weight**	0.44	0.04 to 0.82	**0.04**	0.50	0.11 to 0.89	**0.02**
**Classification 3cp**	12.14	5.51 to 18.86	**0.001**	12.36	5.36 to 19.36	**0.001**
**Classification 4c**	1.76	-8.41 to 9.11	0.69	0.35	-8.41 to 9.11	0.94
**Classification 4cp**	8.29	2.98 to 14.90	**0.01**	8.25	1.47 to 15.03	**0.02**
$\sqrt{Sum\;of\;curves}$						
**Interaction group by time**	- 0.40	-0.77 to -0.03	0.046*	- 0.50	-0.84 to -0.16	0.006*
**Group**	0.48	-1.44 to 2.40	0.33	0.83	-0.11 to 1.77	0.09
**Time**	0.27	0.0 to 0.54	0.07	0.25	0.01 to 0.49	**0.046**
**Height**	- 5.09	-1.63 to 11.81	0.14	-5.77	-12.90 to 1.36	0.12
**Weight**	0.10	0.02 to 0.18	**0.01**	0.10	0.02 to 0.17	**0.01**
**Classification 3cp**	1.49	0.27 to 2.70	**0.02**	1.14	-0.15 to 2.43	0.09
**Classification 4c**	-1.17	-2.74 to 0.40	0.15	-1.69	-3.32 to -0.06	0.05
**Classification 4cp**	0.24	-0.92 to 1.40	0.69	-0.04	-1.31 to 1.23	0.95

* Using Holm-Bonferroni sequential correction all of our calculated p-values remained significant.

#### Sum of curves

To meet the normality assumption, the Sum of Curves was transformed to its square root. After adjusting for confounders, the difference between groups in the $\sqrt{\text{Sum of Curves }}$ over time was statistically significant favoring the Schroth group (-0.40°, 95% CI -0.77° to -0.03°, *p* = 0.048). (Table 3). The $\sqrt{\text{Sum of Curves }}$ decreased by -0.13° in the Schroth, and increased by 0.27° in the control group over six months. This difference in square roots of the Sum of Curves between the groups indicate that a patient with characteristics corresponding to the baseline mean Sum of Curves of 51.2° and the selected covariate set will have a Sum of Curves of 49.3° after six months in the Schroth, and a Sum of Curves of 55.1° in the control group. Moreover, the difference between groups increased with severity.

Weight and classification 3cp had significant main effects on the Sum of Curves (*p* = 0.01, *p* = 0.02, respectively), such that the heavier patients and patients classified with 3cp curve type had on average the largest Sum of Curves. (Table 3) No other covariates (age, height, self-efficacy or brace wear) had an important main effect on the outcome.

### Per protocol analysis

#### Largest curve

When only the completers (per protocol) were considered, the difference in Largest Curve between groups at six months was -4.1° (CI -6.5° to -1.7°, *p* = 0.002), which was larger by 0.6° than in the intention-to-treat.

As in the intention-to-treat analysis, the covariate set included height, weight, and curve classification with similar model coefficient values and significance levels (Table 3).

#### Sum of curves

To meet the normality assumption, the Sum of Curves was transformed to its square root. In the analysis of completers, the difference in the transformed Sum of Curves between groups over time was statistically significant favoring the Schroth PSSE group (-0.50°, 95% CI -0.8° to -0.2°, *p* = 0.001). (Table 3) This difference in square roots of the Sum of Curves between the groups indicate that an average patient with a baseline mean Sum of Curves of 51.2° and the selected covariate set will have a Sum of Curves of 47.7° after the 6-month Schroth PSSE intervention, and a Sum of Curves of 54.8° if in the control group. The difference between groups also increased with severity. Again, per protocol effect estimates were larger than in the intention-to-treat analysis.

Weight had significant main effects on the outcome (*p* = 0.01). (Table 3) Interestingly, unlike in the intention-to-treat analysis, here, classification 3cp did not have significant main effect.

No adverse events were reported during the trial. After adjustment of the p-values using Holm-Bonferroni sequential correction, all results in the ITT and per protocol analysis remained significant.

## Discussion

This RCT demonstrated positive effect of Schroth PSSE added to standard of care (observation and bracing) on the Largest Curve and the Sum of Curves in patients with AIS. The positive effect on the Sum of Curves increased with larger baseline Sum of Curves. In the intention-to-treat analysis, after six months, the Largest Curve decreased in the Schroth group by 1.2°, but increased in the control group by 2.3°. The 3.5° (95% CI -5.9° to -1.1°) difference between groups was statistically significant. The Sum of Curves also decreased over time in the Schroth group. The per protocol analyses for both outcomes produced larger differences between the groups (Largest Curve improved by 1.8° in the intervention and deteriorated by 2.3° in the control group), suggesting that compliance plays a significant role in reaching better outcomes.

Many clinicians and researchers consider a 5° change in Cobb angle clinically important.[42] This threshold is based on reported standard errors of measurement (SEM) for manual Cobb angle measurements. The SEM for our semi-automated method is <2.5°.[34] According to natural history, scoliosis curves progress on average by 0.9°/month, with a range of 0.3° to 1.6°/month.[43] This corresponds to an average expected progression of 5.4° over six months (range 1.8°-9.6°). Bracing was recently reported effective at preventing progression to the surgical range (defined as ≥50°), but did not produce curve improvements on average.[44] In our trial, 17 participants per group wore a brace. Therefore, the difference in Largest Curve change between the groups (3.5°), which was beyond the SEM, together with documented bracing effect[44] after only six months seem clinically important.

Assuming that all patients with missing values experienced curve progression (the worst case scenario), three (12%) deteriorated by >5° in the Schroth group, four improved (16%), and 18 remained stable (72%). In the control group, 10 deteriorated (40%), one improved (4%) and 14 (56%) remained stable (Table 4). If we define a successful treatment as improving curves beyond or remaining within 5° of baseline values, there were 22 (88%) patients who were successfully treated (improved + stable) in the Schroth as compared to 15 (60%) in the control group (Table 4). These results clearly demonstrate the clinical importance of the short-term effects of the Schroth PSSE intervention added to standard of care (observation or bracing) in patients with AIS with curves ≤45°.

**Table 4:** Number of patients with improved, deteriorated and stable curves using a 5° Cobb angle clinical significance threshold.

	Schroth + standard of care	Standard of care
**Deteriorated (Cobb angle increased by ≥5°), number (%)**	3 (12)	10 (40)
**Improved (Cobb angle reduced by ≥5°), number (%)**	4 (16)	1 (4)
**Stable (Cobb angle change <5°), number (%)**	18 (72)	14 (56)

Our results are in line with results of the recent Kuru et al RCT investigating the short-term effect of supervised and non-supervised Schroth PSSE and no intervention on change in the Cobb angle, trunk rotation, height of the rib hump, waist asymmetry and SRS-23 domains in 45 patients with AIS.[31] After 24 weeks, the Cobb angle of the supervised Schroth group improved by 2.5°, and deteriorated by 3.3° and 3.1° in the home exercise and control groups, respectively after six months. Differences between the supervised group and the other two groups were statistically significant. The supervised Schroth intervention was also superior in improving all other measured outcomes. The supervised Schroth intervention consisted of three supervised 1.5 hour-long sessions per week with a Schroth therapist for six weeks (18 sessions in total), after which patients were asked to continue with the treatment at home until six months. The unsupervised exercise group learned the exercises over 1–3 sessions, and then continued on their own at home. Controls received no treatment. This protocol is slightly different from ours despite equal supervised time provided (27 hours). Over six months, we provided five one-hour long treatments during the first two weeks followed by weekly one-hour long sessions (about 27 supervised sessions, 27 hours), compared to 18 1.5-hour long sessions during the first six weeks in Kuru et al’s study (18 supervised sessions, 27 hours).[31] In their RCT, patients were supervised only for six weeks, while in the present RCT weekly supervision continued until the end of six months. In addition, our sample included patients with slightly smaller curves on average (27°-29° in ours vs. 30°-33° depending on groups), of whom 17 per group wore braces compared to none in Kuru et al’s study. Nevertheless, both studies concluded that the supervised Schroth PSSE intervention is similarly effective in decreasing the Cobb angles in patients with AIS while other groups experienced some progression.

Several controlled studies on PSSE for scoliosis have also reported significant effects on curve severity. Wan et al’s short-term RCT reported larger Cobb angle improvements (from 26°±12° to 10°±7°) in the group treated daily with PSSE added to standard of care (surface electrical stimulation, traction and postural training) than with standard of care alone over six months (from 25°±13 to 18°±9°).[45] Comparison with our results is difficult because the standard of care is different and the type of scoliosis investigated in this RCT is unclear.

Monticone et al’s long-term RCT found that PSSE consisting of active self-correction and task-oriented exercises, consistent with Scientific Exercise Approach to Scoliosis (SEAS)[46,47] improved Cobb angles by 5.3° at skeletal maturity in patients with AIS, while traditional exercises were associated with stable curves.[48] None of the patients in their study wore a brace, which explains the smaller curves at baseline in their sample than in ours (19.3°±3.9° vs. 28.5°±8.8°, respectively). Their sample also initially included patients of lower age (12.5±1.1 vs. 13.4±1.6), and Risser grades (0.55 vs. 1.60). According to Lonstein and Carlson’s formula ((Cobb– 3 x Risser)/Age)[4], Monticone’s sample had a 35% risk of progression vs. 65% in our study. Only patients with mild curves and not meeting SRS bracing criteria were included in Monticone’s RCT and followed-up until maturity, which could explain the larger Cobb angle improvement observed compared to our study.

Negrini et al’s[49] prospective study found that one year of using PSSE consistent with the Scientific Exercises Approach to Scoliosis (SEAS) improved the Largest Curve by 0.33°, and the Sum of Curves by 0.67° while in the “usual” rehabilitation program the Largest Curve worsened by 1.12° and the Sum of Curves by 1.38°. Their sample included immature patients (Risser sign 0 to 2) with AIS not meeting bracing criteria, and with baseline Cobb angle of 15°±6°. Our larger improvements compared to Negrini et al’s possibly arose because of a difference in standard of care (observation or bracing versus usual exercise treatment) and our more intense therapy (daily home sessions and weekly visits over six months versus twice/week of home exercises and 4–6 visits over one year).

Noh et al’s short-term retrospective study found better effects on the Cobb angle using PSSE based on a “3D corrective technique” including Schroth and stabilization exercises compared to symmetrical stretching and stabilization exercises.[50] Treatment dose (60-minute sessions, 2–3 times a week, for 30 sessions over four months) was lower than in the present study. Authors reported improvement in Cobb angle of 8.1°±4.5° in the experimental and 4.3°±2.1° in the control group, which were larger than in our study. However, their sample had lower (10%) estimated risk of progression[4] compared to ours (65%).

Otman et al’s prospective one-year short-term uncontrolled cohort study that focused on PSSE consistent with Schroth exercises[28] showed improved Cobb angles in 49/50 adolescents and one stable curve after one year. Treatment was intensive consisting of four-hour sessions, five days/week for six weeks, followed by the same program at home with biweekly follow-ups until six months, and then bimonthly until one year. None of the patients wore brace. Mean Largest Curve decreased from 26.1° to 19.2° over six months. The higher intensity might explain the lower compliance (74%) and high dropout rate (25%), but also a larger Cobb angle change among completers as compared to our study.

Among studies that examined the effect of PSSE, ours was the only one that included patients wearing braces. All, except one prior PSSE study, were with short-term follow-ups ranging from four months to one year. As described by Stokes, lateral curvature of the spine can produce asymmetrical spinal loading resulting in differences in bone growth rates within an individual vertebra. [51,52] This leads to a self-perpetuating progressive deformity during skeletal growth, known as “vicious cycle”. [53] The goal of PSSE schools is to teach patients auto-corrected posture, to stabilize it and integrate in daily life. Auto-correction, defined as the ability to reduce the spinal deformity through the active postural realignment of the spine in three dimensions,[14] balances loads on the convex and concave side of the growing spine and may reverse the “vicious cycle”.[54] There are several PSSE approaches described in the literature with documented evidence of effects on curves including Dobomed, Functional Individual Therapy of Scoliosis (FITS), Lyon method, Schroth, SEAS and Side-Shift. All follow the same principles, although their specific techniques differ. The results of our study strengthen the emerging positive evidence of the effect of PSSE on the Cobb angle change in patients with AIS.

### Strengths and limitations

Several features of this RCT helped reduce the risk of bias. Randomization balanced number of patients wearing a brace in both groups, and curve types distribution. The evaluators and statistician were blinded. We standardized curve classification[55] and exercise prescriptions[56] using algorithms. Patients reported not using co-interventions at follow-ups. Exercise dosage led to high adherence monitored via patients/parent/therapist logbook to minimize the overestimation. Compliance and attendance rates were reported using intention-to-treat. We reported intention-to-treat analysis and acknowledged reasons for missing data (Fig 1). The main reason for non-compliance and dropout was “time constraint due to homework”.

Ours was the first study to stratify randomization by curve types. Patients with major thoracic curves and deviated pelvis to the thoracic concavity (3cp) had the largest curve magnitudes, possibly because of their worst prognosis for progression.[57] In contrast, patients with double major curves (corresponding to 4c) had the smallest curve magnitude all including left lumbar and right thoracic, which have a better prognosis.[57] Differences between patterns emphasize the importance of accounting for curve type in randomization.

A limitation of this study includes possibly limited statistical power due to early termination, and the 12% attrition rate. Regardless, we detected large effects amongst patients (Cohen’s d of 0.92 and 0.77 for change in Largest Curve and Sum of Curves, respectively).

Subject heterogeneity could be another limitation. The selection criteria in our study were wide (heterogeneous) and included all patients with AIS, with all curve types who were undergoing a non-surgical (observation or bracing) standard treatment for scoliosis, because we aimed to generalize the results to the entire population, rather than focus on a very narrow sample. For example, if we were limiting our criteria to only patients who were being monitored and deemed at a high risk of progression, we would not be able to determine the effect of combined bracing with exercises, which was thought to have best outcomes. The purpose of this study was to investigate the effect of enhanced non-surgical (monitoring or bracing) treatment for AIS. Despite the wide selection criteria, we had a balanced representation of the baseline characteristics in the Schroth and control groups in terms of age (13.5 vs. 13.3), height (1.6 vs. 16), weight (45.9 vs. 50.5), curve magnitudes (29.1° vs. 27.9°), number of braced patients (17 vs. 17), Risser sign (1.76 vs. 1.44) and Lonstein-Carlson risk of progression (65% vs. 65%). However, the overall sample of eligible patients had slightly different characteristics: age of 14.2±1.8, height of 1.62 ±9.9m, weight of 53.0±13.3kg, largest curve of 26.0±9.6, Risser of 3.02±1.8, and the risk of progression of about 30%, suggesting that patients who are at higher risk of progression and in the ranges of curves for non-surgical treatment are more interested in applying exercises as an ad on to standard of care. This might be due to the fact that the patients were approached to be part of the study shortly after being seen by a practicing scoliosis surgeon, and the patients’ decision might have been influenced by their surgeon’s opinion about their condition. Nevertheless, exercises should be considered as an ad on to standard of care for scoliosis, because: 1) the patients and their parents are interested in more comprehensive and proactive management, and because 2) the patients are shown to be compliant with the exercises and have better short term outcomes compared to the ones who only receive standard of care.

We included patients of all maturity levels. More mature patients (Risser 3–5) have lower risk of progression, and potentially better treatment success. Nevertheless, our sample’s estimated risk of progression was higher than in most exercise trials. While conducting subgroup analysis on patients with high (Risser 0 to 2) versus low risk of progression (Risser 3 to 5) is warranted, our sample size does not yet allow for this comparison. Most of our patients, however, were deemed at high risk of progression with the distribution of Risser signs presenting as follows: one patient with Risser 5, 11 with Risser 4, five with Risser 3 compared to six patients with Risser 2, four with Risser 1 and 23 with Risser 0. We have made our data available with this publication; future individual patient data meta-analyses will allow for answering such important clinical questions.

The relatively short 6-month follow-up is another potential limitation of the study. Consensus between SRS and SOSORT non-operative management committee published after we underwent the present study recommends a minimum one-year follow-up.[9] However, we believe that if we did not first show at least a small effect at six months, then a study with a later endpoint would not be necessary. The exercise treatment requires daily purposeful commitment and dedication of the patients and their parents, as well as adjustment of their daily routine. Shorter follow-up can assure tangible and meaningful feedback to the patients and promote motivation for continuation of this demanding treatment. In addition, this shorter trial allowed for a stricter control of the intervention, including absence of co-interventions and high compliance, which would be more likely to occur over a longer follow-up. Although, the evidence suggests that long-term PSSE intervention until maturity leads to improvement of the curves[48], it is possible for the curves to deteriorate after a shorter follow-up despite the initial improvement. While the shorter 6-month follow-up allowed for more control over the trial’s possible confounders (i.e., compliance, co-intervention, standardized treatment), it also limited extrapolation of the results to the longer follow-up, at the end of skeletal maturity.

Lastly, this study could not determine the effect of only Schroth exercises, because exercises were combined with standard of care. In order to determine this, our study would have to randomize patients meeting the brace prescription criteria into an exercise only and a brace only group. Ethically, we could not withhold the bracing from the patients meeting the SRS criteria. We aimed to determine the effect of the Schroth PSSE as an add-on to the standard of care, and not as a stand-alone therapy. Since in North America standard of care includes observation and braces for patients with curves ≤45°, our sample consisted of an experimental group of patients who received Schroth + observation or Schroth + brace, and controls, who received only observation or only brace. The proportions of patients receiving observation and bracing within each group was balanced between groups. Wearing a brace was further controlled for in the analyses. The covariate representing the bracing effect was not retained in the statistical models showing that an adjustment for differential effects of bracing in the groups was not required. While it would be interesting to examine the differences between the mentioned subgroups using a factorial design, our sample size did not permit it. The continuing multicenter SETS trial should allow comparisons of the following subgroups: observation versus Schroth PSSE, observation versus Schroth PSSE + brace, brace versus Schroth, as well as brace versus Schroth PSS + brace.

### Future research

Future research investigating the effect of Schroth intervention should include a larger population of patients with AIS, which justifies our multicentre RCT. It is also necessary to establish guidelines for the assessment of the exercise effectiveness, as has been proposed for braces.[42] That way the comparisons between similar exercises trials would be easier and more valid. Future work should highlight the cost-benefit of this promising conservative treatment for scoliosis before a widespread change in practice is implemented. Finally, to avoid the overtreatment, a clinical prediction rule to identify patients who would benefit from the Schroth treatment is also an important step.

## Conclusion

In conclusion, based on both the intention-to-treat and the per protocol analysis, six-months of Schroth PSSE added to standard of care improved curve severity in adolescents with idiopathic scoliosis compared to standard of care. The completers experienced larger benefits from the intervention compared to the entire sample, which emphasizes the importance of compliance with the exercise program. Low dropout and adequate compliance rates indicate the feasibility of adding Schroth intervention to the standard of care in North America. This trial increases the level of evidence on the short-term benefits of Schroth PSSE for AIS by its methodological rigor and justifies continued investigation in the ongoing Multicenter Trial, in which we will also identify which children are most likely to benefit from Schroth exercises.

## Supporting Information

S1 Fig CONSORT 2010 Compliance Checklist. (DOCX)

S1 File Full study protocol approved by our local Human Research Ethics Board Biomedical. (PDF)

S2 File Excel file containing study data. (XLSX)
