# Supplementary material for: Schroth Physiotherapeutic Scoliosis-Specific Exercises Added to the Standard of Care Lead to Better Cobb Angle Outcomes in Adolescents with Idiopathic Scoliosis – an Assessor and Statistician Blinded Randomized Controlled Trial
Source: PLoS One. 2016 Dec 29;11(12):e0168746. doi: 10.1371/journal.pone.0168746 (PMC5198985; doi:10.1371/journal.pone.0168746)
Supplement: S1 File — (PDF) [file pone.0168746.s002.pdf]

Date: 1/15/2016, 4:11:40 PM

Print Close

ID: Pro00011552

View: 1.1 Study Identification

Status: Approved

ID:Pro00011552

Status: Approved

1.1 Study Identification

*All questions preceded by a red asterisk * are required fields. Other fields may be required by the REB in order to evaluate your application.*

Please answer all presented questions that will reasonably help to describe your study or proposed research.

1.0 * **Short Study Title** *(restricted to 250 characters)*:
SETS study

2.0 * **Complete Study Title** *(can be exactly the same as short title)*:
Schroth Exercise Trial for Scoliosis

3.0 * **Select the appropriate Research Ethics Board** (*Detailed descriptions are available by clicking the HELP link in the upper right hand corner of your screen*):
HREB Biomedical

4.0 * **Which office requires notification of ethics approval to release funds or finalize the study contract?** *(It is the PI's responsibility to provide ethics approval notification to any office other than the ones listed below)*
University of Alberta - Research Services Office (RSO)

5.0 * **Name of Principal Investigator** *(at the University of Alberta, Covenant Health, or Alberta Health Services)*:
Eric Parent

6.0 **Investigator's Supervisor** *(Required for graduate students and trainees NOT applying to the Health Research Ethics Board (HREB). The HREBs do not accept graduate students or trainees as Principal Investigators in an ethics application. Please enter your supervisor as the PI and yourself as a co-investigator in your application for HREB.*

Supervisor is required if the PI is a student researcher.

7.0 * **Type of research/study:**
Graduate Student - Thesis, Dissertation, Capping Project

8.0 **Study Coordinators or Research Assistants:** People listed here can edit this application and will receive all HERO notifications for the study:

Name	Employer
Kathleen Shearer	MH Surgery
Sanja Bosnjak	RM Dean Rehab Medicine
Elise Watkins	Student

9.0 **Co-Investigators:** People listed here can edit this application but do not receive HERO notifications unless they are added to the study email list:

Name	Employer
Marc Moreau	TEMP
Douglas Hill	MH Surgery

10.0 **Study Team** *(Co-investigators, supervising team, other study team members):* People listed here cannot edit this application and do not receive HERO notifications:

Last Name	First Name	Organization	Role	Phone	Email
Hedden	Doug	University of Alberta, dept. Surgery	Chair	780-407-7002	doug.hedden@albertahealthservices.ca
Mahood	Jim	Stollery Hospital	Edmonton Scoliosis clinic surgeon	780-455-5115	jkmahood@hotmail.com
Moreau	Alain	Hopital Ste-Justine	Director of research center/ blood test analyses	514-345-4931 poste: 3476	alain.moreau@recherche-ste-justine.qc.ca
Southon	Sarah	University of Alberta/Stollery Hospital /Edmonton Scoliosis clinic	Nurse Practitioner/ eligibility	780-407-1560	Sarah.southon@albertahealthservices.ca

ID: Pro00011552

View: 1.2 Additional Approval

Status: Approved

1.2 Additional Approval

1.0 * **Departmental Review:**
RM Physical Therapy

2.0 **Internal Review:**

ID: Pro00011552

View: 1.3 Funding Information

Status: Approved

1.3 Study Funding Information

1.0 * **Type of Funding:**
Grant (external to the institution)
If OTHER, provide details:

Your answer here will deteremine the next form you will see.

Funding Source

2.0 **2.1 Select all sources of funding from the list below:**

Glenrose Rehabilitation Hospital	GRH
Scoliosis Research Society	SCOL

2.2 If not available in the list above, write the Sponsor/Agency name(s) in full (you may add multiple funding sources):
There are no items to display

3.0 **Location of funding source** *(required if study is funded)***:**
Canada
US

RSO University-Managed Funding

4.0 **4.1 If your funds are managed by the Research Service Office (RSO), select the project ID and title from the lists below to facilitate release of your study funds.** *(Not available yet)*

4.2 If not available above, provide all identifying information about the study funding:

	Project ID	Project Title	Speed Code	Other Information
View	RES0004194	A Pilot Study for Randomized Controlled Trial to Develop a Prediction Rule to Identify Patients with Adolescent Idiopathic Scoliosis who will Respond to Schroth Exercises	56294	

ID: Pro00011552
Status: Approved

View: 1.4 Conflict of Interest

1.4 Conflict of Interest

1.0 * **Are any of the investigators or their immediate family receiving any personal remuneration (including investigator payments and recruitment incentives but excluding trainee remuneration or graduate student stipends) from the funding of this study that is not accounted for in the study budget?**
Yes **No**

If YES, explain:

2.0 * **Do any of investigators or their immediate family have any proprietary interests in the product under study or the outcome of the research including patents, trademarks, copyrights, and licensing agreements?**
Yes No

3.0 **Is there any compensation for this study that is affected by the study outcome?**
Yes **No**

4.0 **Do any of the investigators or their immediate family have equity interest in the sponsoring company? (This does not include Mutual Funds)**
Yes **No**

5.0 **Do any of the investigators or their immediate family receive payments of other sorts, from this sponsor (i.e. grants, compensation in the form of equipment or supplies, retainers for ongoing consultation and honoraria)?**
Yes No

6.0 **Are any of the investigators or their immediate family, members of the sponsor�s Board of Directors, Scientific Advisory Panel or comparable body?**
Yes No

7.0 **Do you have any other relationship, financial or non-financial, that, if not disclosed, could be construed as a conflict of interest?**
Yes **No**

If YES, explain:
2.0

Ste-Justine University Hospital holds 2 patents and others patents are pending resulting from the discoveries of Dr. Alain Moreau. In addition, Ste-Justine University Hospital has signed a licensing agreement with Paradigm Spine LLC (New York City, USA) for the commercialization of all Dr. Moreau�s diagnostic tests and other innovations in the field of scoliosis.

5.0

Dr. Moreau has received 2 research grants from Paradigm Spine LLC, which cover in part the cost of the tests during the clinical validation of the tests in Canada and worldwide. Additional funding is provided by a research grant from The Yves Cotrel Foundation, Institut de France (Paris, France). Dr. Moreau reported that he has received consulting fees from Paradigm Spine LLC.

6.0

Dr. Moreau is serving on the scientific advisory panel of Paradigm Spine LLC.

Important
If you answered YES to any of the questions above, you may be contacted by the REB for more information or asked to submit a Conflict of Interest Declaration.

ID: Pro00011552 View: 1.5 Study Locations and Sites
Status: Approved

1.5 Research Locations and Sites

* **List the locations of the proposed research, including recruitment activities. Provide name of institution or organization, town, or province as applicable** (e.g. On campus, Alberta public elementary schools, shopping malls, doctors' offices in Lesser Slave Lake and Lac La Biche, AHS facilities in Zone 5, post-secondary students at UBC, UA, UT, McGill and Dalhousie, internet websites, etc.)**:**

1.0 University of Alberta Hospital - Scoliosis Clinic (Recruitment, and initial eligibility assessment)
Canadian Blood Service Repository (blood Processing in medical science building) and NACTRC blood collection by Canadian blood service repository (Campus plaza)
University of Alberta- Clinical Sciences Building, room 8-110K Scoliosis Surface Topography Lab
University of Alberta - Rehabilitation Sciences - Corbett Hall, room 3-78, Principal Investigator's Lab(Assessments, treatment)

* **Indicate if the study will utilize or access facilities, programmes, resources, staff, students, specimens, patients or their records, at any of the sites affiliated wiht the following** *(select all that apply):*
Alberta Health Services

List all facilities or institutions as applicable:

2.0 Recruitment will be done via the Edmonton Scoliosis Clinic at the Glenrose Rehabilitation Hospital and Stollery Hospital (Radiographs and Quality of Life questionnaires will be extracted from the clinical databases).
Blood will be extracted by Canadin Blood Repository research nurse in Campus Plaza for Blood Extraction and shipped for preparation of samples to the Canadian Blood service repository in Medical Science building and then shipped for analysis to the Hopital Ste-Justine in Montr�al.
Surface topography scans will be recorded at the Clinical Sciences Building, room 8-110K Scoliosis Surface Topography Lab, affiliated with the Stollery's Edmonton Scoliosis Clinic.

If the study involves researchers in other institution(s), will ethics approval be sought from other institutions/organizations *(eg. another university, Alberta Cancer Board, school district board, etc)***?**
No

3.0 **If YES, provide a list:**

Name
There are no items to display

ID: Pro00011552 View: 2.1 Study Objectives and Design
Status: Approved

2.1 Study Objectives and Design

1.0 **Proposed Start Date:**
9/14/2010

2.0 **Date that you expect to start working with human participants:**
9/14/2010

3.0 **Date that you expect to finish working with human participants:**
9/14/2012

* **Provide a lay summary of your proposed research suitable for the general public** *(restricted to approx. 300 words)*. **If the PI is not affiliated with the University of Alberta, Alberta Health Services or Covenant Health, please provide institutional affiliation:**

Summary
Adolescent Idiopathic Scoliosis (AIS), a 3D spinal deformity, is the most common orthopedic condition in adolescents and affects mostly females. Scoliosis reduces quality-of-life, often results in pain, poor function and self-image. Schroth method utilizes asymmetric 3D endurance, strength and special breathing exercises aiming to correct posture, scoliotic curves and breathing pattern.
A systematic review on scoliosis exercises found that assymetric exercises slowed the worsening of scoliosis. Many cohort studies showed that Schroth method could be effective in slowing the curve progression, as well as improving the scoliotic curves. Slowing scoliosis progression may help prevent invasive surgery and bracing.
4.0 However, history showed that not all patients benefited from Schroth exercises. A prediction rule will help therapist offer exercises only to patients likely to benefit. A strong RCT is needed to confirm results from cohort studies and to identify the predictors of success in patients with AIS treated with Schroth exercises.

One hundred patients with juvenile idiopathic scoliosis (JIS), AIS, or syringomyelia without chiari , curves 10°-45°, between the ages of 10-18, with all Schroth curve types (3c, 3cp, 4c and 4cp), will be recruited from our clinic. They will be randomized to observation/bracing(as the current treatment standard dictates) or Schroth exercises (alone or with brace). History, questionnaires, physical, blood and surface topography assessments will be done at baseline, 3 and 6 months. Radiographs and SRS-22 questionnaires will be extracted from the Clinic database, at baseline, 6 and 12 months and every 6 months until discharge from the scoliosis clinic. The control group will receive no treatment. The Schroth exercise program will consist of 5 individual sessions, followed by weekly group sessions and a home exercise program for 6 months. A certified Schroth therapist will prescribe exercises using an algorithm. After 6 months control subjects will crossover to the control group. Recruitment, eligibility, compliance, and drop out rate, along with effect sizes for all outcomes will be determined. Ultimately, a prediction rule to guide prescription will be created.

* **Provide a description of your research proposal including study objectives, background, scope, methods, procedures, etc.** *(restricted to approx. 1,000 words)*. **Footnotes and references should not be included here. Research methods questions in Section 5 of this form will prompt additional questions and information.**

Schroth Exercise Trial for Scoliosis
Background
Adolescent Idiopathic Scoliosis (AIS), a 3D spinal deformity, is the most common orthopedic condition in adolescents and affects

mostly females. In mild scoliosis from 10° to 25° the ratio is 4:1, in moderate AIS from 25° to 50° the ratio is 7:1, while in severe AIS >50° the ratio is 10:1. Scoliosis reduces quality-of-life, often results in pain, poor function and self-image.

Usually, curves between 25-45° are prescribed a brace and curves over 45° are treated surgically. Smaller curves are not treated. Schroth method utilizes asymmetric 3D endurance, strength and special breathing exercises aiming to correct posture, scoliotic curves and breathing pattern. Schroth therapists provide support by skillful resistances, auxiliary handholds, and specific verbal instructions and feedback from mirrors. Schroth exercises aim to restore proper physiological three-dimensional alignment.

Schroth treatment has proven effective in improving strength, posture, slowing progression and improving Cobb angle in many cohort studies. However, these results have not yet been tested in RCTs.

Objectives

To conduct a pilot study for an RCT on the effect of Schroth exercises for AIS compared to the current treatment standard (observation or bracing prescribed as per SRS guidelines). The pilot aims (calculation after 20 subjects) to determine:

1) The recruitment and eligibility rate for the RCT;
2) The percent compliance with exercises and drop out rate;
3) The effect size for all outcomes; and
4) The predictors of success in patients with AIS treated with Schroth exercises.

Objectives of the RCT are to:

(1) Create a prediction rule to guide prescription;
(2) Determine the effects of Schroth exercises on spinal deformity and quality-of-life.

Methods

Subjects

Patients with AIS, JIS, or syringomyelia without chiari (n=100) will be recruited. Inclusion criteria are: (1) curves 10°-45°, (2) age 10-18 (3) All Schroth curve types (3c, 3cp, 4c and 4cp), (4) treated with or without brace (5) living near Edmonton. Those planning or post-surgery will be excluded.

Procedures

At routine scoliosis clinic visits a nurse will determine eligibility and randomize participants to the observation (control) or the Schroth exercise group. Outcomes will be measured at baseline, 3 and 6 months. Primary outcomes include baseline and 6-month radiographic curve measurements and SRS-22 questionnaire. Secondary outcomes will include postural measures (Surface Topography), Global Rating of Change (GRC), Self-efficacy questionnaire, pain, progression risk (blood test) and back muscle endurance. Predictors of success will be collected at baseline.

Controls will receive current standard of treatment for 6 months (Observation or brace prescribed as per SRS guidelines). Patients treated wtih Schroth exercises will weekly participate in supervised Schroth exercises and complete a daily home program for 6 months. The baseline and 6-month exam will be completed within 2 weeks of a scoliosis clinic visit with a follow-up at 3 months. After 6 months, controls will crossover to the treatment group. After 12 months and for every scoliosis visit afterwards until discharge, we will also extract the radiograph and SRS-22 questionnaire from the Edmonton Scoliosis records

Schroth exercises:

Patients for which the SRS guidelines would recommend observation as standard of care will do exercises alone. Patients for which the SRS guidelins would recommend bracing will do exercises in addition to being prescribed a brace.

Private sessions- During the first 2 weeks, patients will attend 5 1-hour-long individual sessions to learn Schroth principles, exercises and correct breathing, and to confirm independent adequate performance with a checklist.

Home exercises- A 30-min. daily home exercise program of 3 to 4 exercises will be adjusted during the first 3 months using an algorithm. Compliance will be monitored using a logbook for 6 months. Completion exercises during the last 12 months will also be monitored using a logbook.

Group Sessions- Classes increase compliance. Patients will attend weekly 1-hour-long group therapist-led exercise classes. Adequate exercise performance will be assessed using the checklist. Pain will be monitored.

5.0 *Algorithm* – An algorithm guides exercise prescription, intensity and progression from static to dynamic execution. Prescription begins with easier exercise. If performed adequately, a more challenging exercise will be tried. If performance is inadequate, an easier exercise will be attempted. Exercises performed adequately will be prescribed.

At follow-up, if performance is adequate, dosage will increase to target intensity. Target intensity increases repetitions aimed to enhance endurance. Final tension intensity is nearly maximum isometric contraction held in corrected posture.

Controls: Controls will receive the standard of care appropriate for their curves (observation or bracing prescribed as per SRS guidelines), and will complete baseline, 3 and 6 months exams. After 6 months they will cross-over to the treatment group.

Questionnaires:

Demographic/History- Socio-demographics, symptoms history and modifying factors will be self-reported.

Pain rating and diagram- A numeric scale from 0 (no pain) to 10 (worst imaginable pain) will assess current, best, worst intensity in the last 24 hours. A diagram will assess location of symptoms.

Scoliosis-Research-Society questionnaire (SRS-22r)- The SRS-22r assesses quality-of-life within 5 domains: function, pain, self-image, mental health (5 questions each), and satisfaction (2 questions).

3D Physical activity recall (3DPAR)- quantifies habitual physical activity chosen among 55 activities listed, and their intensities for blocks of 30-min over 3 days. The 3DPAR score is determined using the metabolic equivalent (MET) levels.

Walter Reed Visual Assessment Scale- includes 7 items pertaining to the deformity (spinal deformity, rib prominence, lumbar prominence, thoracic deformity, trunk imbalance, shoulder asymmetry and scapular asymmetry) presented by drawings of 5 levels of deformity severity.

Self-efficacy – measures self-efficacy for overcoming barriers to physical activity using 8 items rated from 1 (Disagree a lot) to 5 (Agree a lot).

Global Rating of Change (GRC)- A 15-point GRC ranging from –7 (very great deal worse) to +7 (very great deal better) will be used.

Physical Exam*(Appendix E)*: An physiotherapist blind to groupings will assess curve type and hip range-of-motion in flexion, extension, internal rotation, the straight-leg-raise and FABERE tests. Sorensen's test will assess back extensor muscle endurance. Lumbar (inclinometers at L5/S1 and T12/L1) and thoracic (C7/T1 and T12/L1) spinal range-of-motion will be recorded: lumbar flexion, extension and side flexions. Scoliometer will record asymmetry during the Adam bending test.

Radiographs- Standing postero-anterior radiographs in a positioning frame at baseline, 6 and 12 months and from every scoliosis clinic visits until discharge will be used to measure the Cobb angle, rotations, apex translation. An experienced evaluator blind to group assignment will extract measures.

Blood test - At baseline, 3 and 6months, 10ml of blood will be extracted at the Campus Plaza by a Blood Service Repository research nurse, and shipped to Dr. Moreau's laboratory to quantify the SCD44 and OPN levels that indicate the progression risk.
Surface topography (ST) – An experienced evaluator will operate four scanners and use a positioning frame to scan torso shape. The following ST parameters will be extracted by digitizing 15 points on de-identified scans (blinding): cosmetic score, shoulder angle, scapula angle, waist asymmetry, trunk rotation, kyphotic and lumbar indices.

Analyses:
Completed after first 20 subjects:
1: Recruitment rate is the percentage of patients enrolled out of all patients invited. Eligibility rate is the percentage of patients meeting selection criteria out of all assessed.
2: Compliance is the percentage of visits attended and percentage of prescribed home exercises reported performed. Drop out rate will be calculated.
3: Effect sizes will be estimated for all outcomes to guide sample size estimations for future studies.
For the whole trial.
1) Prediction rule development
2)For effect determination
Descriptive statistics will be used to compare groups at baseline. Intention to treat ANCOVA will be used to compare treatment groups and to determine the effect of the interventions over time while controlling for curve severity, age, and pain ratings at baseline. Feasibility:

Two PhD students are certified Schroth therapists. Six patients/month meet selection criteria. Assuming ~28% enrollment, the study will complete within 5 year. We have research experience with the assessments and exercise studies for spinal problems. We have equipment and space. Staff and patients expressed interest for Schroth exercises.

Subjects classified as successful (improvement by 5 degrees on radiograph or by 2XMCID on SRS-22). Predictors of success will be identified first by univariate methods (T-test/ROC curves for continuous and Chi-Square for dichotomous predictors). Then univariate predictors will be tested for whether they are effect modifiers or simple prognostic factors. In ANOVA analyses, we will examine if the following interaction term reaches significance: Treatment group X Time X Status on the potential predictor. Finally, using stepwise logistic regression, the best combination of variables predicting a good response to the treatment will be selected to enter into the prediction rule.

Describe procedures, treatment, or activities that are above or in addition to standard practices in this study area *(eg. extra medical or health-related procedures, curriculum enhancements, extra follow-up, etc)***:**
Surface topography and radiograph scanning (which will be extracted from the hospital database) are standard practices at the Edmonton Scoliosis Clinic. SRS-22 and Walter Reed questionnaires are current practice as well. The data routinely collected at scoliosis clinic visits will be used in the study. Clinics are routinely schedule every 6 months for patients meeting the eligibility criteria in our study. Our 3 months assessment and is additional to the routine visits. Some tests at baseline, 3 and 6 months are additional to routine visits and all exercise treatments are additional to routine visits.

6.0 Participating in the study requires completing tests and questionnaires in addition to usual procedures. Patients with scoliosis, participating in this study, will be asked to also give blood at baseline, 3 and 6 months visits, for the diagnostic and prognostics of scoliosis purposes, as this is not part of routine care. Physical examination at baseline, 3 and 6 months including range of motion of back and lower extremity are not current practice. The following questionnaires completed at baseline, 3 and 6 months are extra procedures, too: Demographic/History, Pain rating and diagram, 3D Physical activity recall, Self-efficacy and Global Rating of Change.

All exercise visits are additional to routine scoliosis visits.

If this research proposal has received independent scientific or methodological review, provide information *(eg. names of committees or individuals involved in the review, whether review is in process or completed, etc)***:**
7.0 Glenrose Hospital Foundation,Clinical Research Grant Review Committee (completed)
Scoliosis Research Society, Small Exploratory Grant Review Committee (completed 2009 and 2010)
Women's and Children Health Research Institute, CIHR Pre-review Process (completed)

If this application is related to or builds upon a previously approved application at the University of Alberta, please provide the study title and ethics file/approval number or any other reference if available:
- Blood test procedure use in the study has been approved in the study: "Spinal Stiffness in Adolescents with Idiopathic Scoliosis" (PRO00006957)
8.0 - The surface topography assessment protocol has been used in the study:"Full Torso Surface Topography for Scoliosis" (PRO00006268) and in the study:"360 Degrees Surface Topography Parameters (Norms)" (PRO00003600)
- The use of SRS-22 Questionnaire was approved in all of the above mentioned studies.
- The physical assessment has been approved in the PRO00006957 and in the PRO00003600 studies.
- The radiograph analyses have been approved in the PRO00006268 study.

ID: Pro00011552 View: 3.1 Risk Assessment
Status: Approved

3.1 Risk Assessment

1.0 * **After reviewing the Minimal Risk Criteria provided in User Help, provide your assessment of the risk classification for this study:**
Minimal Risk

2.0 * **In a scale of 0 to 10 where 0 = No Likelihood, 5 = Moderate Likelihood and 10 = Extreme Likelihood, put a numerical rating in response to each of the following:**

Rate	Description of Potential Risks and Discomforts
0	Psychological or emotional manipulations will cause participants to feel demeaned, embarrassed, worried or upset
3	Participants will feel fatigued or stressed
2	Questions will be upsetting to the respondents
0	Participants will be harmed in any way
0	There will be cultural or social risk � for example, possible loss of status, privacy, and/or reputation
3	There will be physical risk or physiological manipulations, including injury, infection, and possible intervention side-effects or complications
3	The risks will be greater than those encountered by the participants in everyday life

* **Provide details of short- and long-term risks and discomforts:**
- For physical assessment:

We do not expect adverse effects to result from the study physical assessment. All the procedures are routinely used in the assessment of patients with scoliosis and are considered non-invasive. In the unlikely event where we would detect a spinal deformity warranting further investigation in the healthy teenagers, we will provide information on the nature of our findings and suggest referral to the patient�s family physician. We do not expect that subjects will suffer any side-effects from the physical assessment in this study. The assessment involves simple movements and tests. Participants should not feel pain in this study. Rarely, participants may feel sore during the first few days following a simple physical assessment. For those experiencing soreness it should disappear in a few days. The tests are not invasive and do not use radiation. There could be other side-effects which it is not possible to anticipate.
- For blood testing:
3.0 Blood testing does not cause problems for most people. However, blood testing may cause a small bleeding or a bruise after testing or the next day. The blood test is still experimental. The blood test is not to diagnose scoliosis. The blood test results in our files have no clinical or diagnostic value.
- For the treatment:
Schroth exercises are designed to improve the ability to maintain correct posture through endurance and strength training. Schroth exercises include de-rotation, de-flexion, and stretching exercises to establish vertebral alignment, torsional respiration exercises, and strengthening exercises for the abdominal, back, leg and foot muscles, and as such are not expected to have any adverse effects on patients. The exercises involve simple movements and positioning. Participants should not feel pain in this study. Rarely, participants may feel sore during the first few days following trying a new exercise. For those experiencing soreness it should disappear in a few days.
- For the surface topography:
The full torso scanning procedure involves removing clothes of the torso, hiding the breast using stick-on bra and having some reference points marking on the skin. Some participants may feel uncomfortable with their torso exposed.
-In both study groups, some patient's scoliosis may progress to the extent where bracing or surgery prescription criteria are met.

* **Describe how you will manage and minimize risks and discomforts, as well as mitigate harm:**

- We do not expect adverse effects to result from the study. All the procedures are routinely used in the assessment of patients with scoliosis and are considered non-invasive. In the unlikely event where we would detect a spinal deformity or a pathology warranting further investigation, we will provide information on the nature of our findings and suggest referral to the patient�s family physicians. We will be available to discuss our findings with the physician at the patient�s request. Testing procedure can be interrupted at the patient's request at anytime.
- Blood testing is done by professionals based on a requisition using routine hospital procedures. Participants can refuse the test if needed. If test results suggest that follow-up is required they will be invited to attend the scoliosis clinic again within six months of the blood draw.
- The surface topography scanning procedures are all done by a female. The subjects will be oriented to the scanning area.
4.0 Participants will be instructed in the use of the stick-on bras. The evaluator will then leave the patient in the closed off scanning area to disrobe privately and apply the bras. The patient will be given a hospital gown for privacy when the evaluator returns to apply the reference marks on the skin and to adjust the positioning frame. The evaluator will then leave again and the patient will disrobe. The surface scan will be obtained immediately. During the scan the subject will be offered to be accompanied by a family member or a friend if they desire. The scanner operator will be a female. The analysis of the scan will be done by a female and the head will be cropped off of the scan to protect patient privacy. Patients may refuse to participate at any point.
- Schroth therapist will individually instruct all the patients in the execution of the exercises in the first 5 sessions, and the group sessions will be led by a Schroth therapist. All exercises can be modified to minimize possible discomfort that can be caused by inadequate level of intensity.
- In the case of patient's scoliosis progression to the extent where bracing or surgery prescription criteria are met, they will be referred to their treating surgeon and prescribed treatment deemed appropriate. SRS prescription criteria will be used.

* **If your study has the potential to identify individuals that are upset, distressed, or disturbed, or individuals warranting medical attention, describe the arrangements made to try to assist these individuals. Explain if no arrangements have been made:**

5.0 In the unlikely event where we would detect a spinal deformity or a pathology warranting further investigation, we will provide information on the nature of our findings and suggest referral to the patient�s treating physicians. We will be available to discuss our findings with the physician at the patient�s request. If the experimental blood test results suggest that progression of scoliosis is likely on the basis of the limited information available we will recommend follow-up of the participant a the Edmonton scoliosis clinic within six months. In the case of patient's scoliosis progression to the extent where bracing or surgery prescription criteria are met, they will be referred to their treating surgeon and prescribed treatment deemed appropriate. SRS prescription criteria will be used. The Clinic coordinator will be notified and a visit scheduled for the participant.

ID: Pro00011552 View: 3.2 Benefits Analysis
Status: Approved

3.2 Benefits Analysis

Describe any potential benefits of the proposed research to the participants. If there are no benefits, state this explicitly:

1.0 There are possible benefits for the participants in this study. There is evidence that suggests Schroth exercises can improve spinal curvature, slow the progression of scoliosis, correct posture, and increase strength in some patients with AIS. The benefits are not guaranteed and patients that have received Schroth exercise treatment in the past have not always had these results. If findings from the evaluations suggest that you need additional medical attention, you will be contacted and referred appropriately.

* **Describe the scientific and/or scholarly benefits of the proposed research:**

2.0 Results will help determine the effects of Schroth exercises on children with AIS. Results will help clinicians determine who benefits from and who should receive this exercise treatment.

Describe any benefits of the proposed research to society:

3.0 - Results of our study will help determine whether this exercise treatment is appropriate to implement at the Edmonton Scoliosis clinic as an alternative to standard practice (observation/ or bracing) for the smaller curves (10-45 degrees according to Cobb).
- Results of our study may also help other clinics in the world to decide whether they want to offer Schroth treatment.

Benefits/Risks Analysis: Describe the relationship of benefits to risk of participation in the research:

4.0 Risks are minimal and controlled. The results could potentially have great benefit for clinical practice by providing evidence for the conservative treatment for adolescent idiopathic scoliosis and assist physicians in prescribing exercises to the right patient.

ID: Pro00011552 View: 4.1 Participant Information
Status: Approved

4.1 Participant Information

Describe and justify the inclusion criteria for participants (eg. age range, health status, gender, etc):

Patients with Adolescent Idiopathic Scoliosis (AIS), with juvenile idiopathic scoliosis (JIS), or syringomyelia without chiari from the Edmonton Scoliosis Clinic (n=100) will be recruited from our scoliosis clinics. AIS is defined as idiopathic scoliosis diagnosed between the ages of 10-18. JIS is defined as idiopathic scoliosis diagnosed between the ages of 5-10. Idioapthic scoliosis may have appeared between the ages of 5-10, but not been diagnosed until after the age of 10. Therefore, including patients with JIS and AIS in the study is not including two distinct forms of scoliosis, rather a continuum of idioapthic scoliosis. Patients with syringomyelia without chiari are common and does not exclude a patient's diagnosis as AIS or JIS.

Both females and males are targeted. Schroth exercises are precribed for both males and females, although females are more frequently severely affected. Other inclusion criteria are:

(1) magnitude of spinal curve 10° to 45° (Cobb angle),

-Larger curves meet the clear indication recommended by the Scoliosis Research Society to be prescribed surgery. Small curves in skeletally immature adolescents have higher risk of progression. Curves over 30° are more likley to progress after skeletal maturity. European consensus for conservative treatment recommends exercises alone below 20° and exercises in combination with a brace for patients with curves greater than 20°.

(2)may be treated with or without brace

-We have broadened inclusion criteria to be inclusive of all conservative treatment offered at the Edmonton scoliosis
1.0 clinic (observation and bracing). European consensus for conservative treatment recommends exercises alone below 20° and exercises in combination with a brace for patients with curves greater than 20°. Including patients with and without brace treatment allows for comparison of exercise alone and in combination with brace treatment. Including brace treatment as a potential predictor of treatment success is representative of decisions clinicians are making in the treatment of patients with scoliosis. We will examine whether bracing in combination with exercises is predictive of success beyond the other treatment combinations.

(3)Risser 0-5 (complete range of skeletal maturity)

-Patients with curves over 30° have a risk of progression beyond skeletal maturity. Schroth exercises are recommended for all curve sizes.

(4) age 10-18

-Patients are treated between the ages of 10-18 at the Edmonton Scoliosis Clinic. Inclusion of patients over the age of 10 allows for comprehension of Schroth exercise program.

(5) ability to travel to U of A at least weekly.

- To ensure that we can assess the efficacy of the proposed exercise protocol we require the participant to attend weekly exercise sessions at Corbett hall. This will ensure feasibility of the trial with opportunity for maximal compliance.

(6)We will recruit 25 patients from each of the 4 Schroth curve types.

-Each of the 4 Schroth curve types is treated with a slightly different exercise protocol. To our knowledge there is no evidence that the Schroth approach works as well for each curve type. To determine the predictive ability of Schroth curve type to predict success with the approach it is necessary to stratify for curve type as the prevalence of each curve type differ.

Describe and justify the exclusion criteria for participants:

(1)Patients with curves >45° may be surgical candidates, and will be excluded.

-Our clinic and the Scoliosis research foundation clearly recommend that patients with such large curves should be considered for surgery. In practice, these patients may be prescribed surgery and not deemed candidate for an exercise program.

(2) Patients who have had surgery will be excluded.
2.0 -Previous surgical intervention could have a confounding effect in the efficacy analysis.

-A separate study could examine the effect of exercises combined with surgical correction but the current study will not address this question.

(3) Patients under the age of 10 will not be included, as the Schroth exercise program is not designed for juveniles. The level of comprehension may be too difficult for ages 5-10 (age of diagnosis with JIS). Patients over the age of 18 are excluded as treatment and observation at the Edmonton Scoliosis clinic is complete at age 18.

3.0 **Are there any direct recruitment activities for this study?**

◉ **Yes** ○ No

Participants

Total number of participants you expect to enroll *(including controls, if applicable)*:

100

4.0 **Of these how many are controls, if applicable** *(Possible answer: Half, Random, Unknown, or an estimate in numbers, etc).*

50

If this is a multi-site study, how many participants *(including controls, if applicable)* **do you anticipate will be enrolled in the entire study?**

Justification for sample size:

- The minimum sample size required to test the overall fit of a regression model is 10 subjects per predictor variable. To derive a clinical prediction rule requires testing the interaction of a large number of baseline variables, which may or may not be entered into the final rule. We are using a two stage process (univariate then multivariate) to reduce the number of variables entered in the regression analysis. In this study, we are limiting the potential prediction variables to under 20, which is more parsimonious than previous CPR derivations. CPRs are not recommended for clinical practice until they are validated in other populations of patients,
5.0 in other settings, with other clinicians. In the subsequent process of validation, the predictors are limited to those in the rule, with opportunity for a study powered to determine significant effect modifiers.
- To detect a 0.50 effect size when comparing the change in the primary outcome in the exercise and control group with 80% power using an unilateral test and an alpha of 0.05 a sample of 50 patients per group will be sufficient. We anticipate a 75% completion rate from the enrolled participants based on previous compliance reports in exercise studies. However, since we are planning intention to treat analysis our sample will remain at 50.

If possible, provide expected start and end date of the recruitment/enrollment period:

6.0 **Expected Start Date: 9/14/2010**

Expected End Date: 9/14/2012

ID: Pro00011552 View: 4.2 Recruit Potential Participants

Status: Approved

4.2 Recruit Potential Participants

Recruitment

1.1 Will potential participants be recruited through pre-existing relationships with researchers *(eg. employees, students, or patients of research team, acquaintances, own children or family members, etc)*?

◉ **Yes** ○ No

1.2 If YES, identify the relationship between the researchers and participants that could compromise the freedom to decline

1.0 **(eg. professor-student). How will you ensure that there is no undue pressure on the potential participants to agree to the study?**
Some of the study team members are involved in the management of patients attending the Edmonton Scoliosis Clinics. Patients with scoliosis from the Edmonton Scoliosis Clinic will be informed of the study and invited to participate by the clinic coordinator when arranging clinic visits. The clinic coordinator will send the study information by email or by phone when booking the clinic visit. The booking is not dependent of patient's agreement to participate. Once informed patients will contact the researcher to learn more about the project and arrange participation in the study. The coordinator will explicitly mention that patient may refuse to participate. The surgeon may answer questions about the project during the patient's visit but will not be directly involved in the enrollment process. The clinic's nurse practitioner will assist in determining patient's eligibility but will not decide if a patient is ultimately eligible or not (this will be confirmed during the baseline evaluation).

2.0 **Outline any other means by which participants could be identified** *(eg. response to advertising such as flyers, posters, ads in newspapers, websites, email, listservs; pre-existing records or existing registries; physician or community organization referrals; longitudinal study, etc)***:**
The prevalence of Scoliosis is relatively low. Many patients with small curves do not know they have scoliosis. Therefore, advertising the study to the public would likely be expensive, require a large effort and may not be be a very effective strategy to reach patients with small scoliosis curves. The majority of patients with scoliosis in the Edmonton area are referred to the Regional Edmonton Scoliosis clinic. Therefore, it is the ideal place to recruit subjects from our study effectively.

ID: Pro00011552 View: 4.3 Recruitment Contact Methods
Status: Approved

4.3 Recruitment Contact Methods

1.0 **How will initial contact be made? Select all that apply:**
Contact will be made through an intermediary

2.0 **If contact will be made through an intermediary** *(including snowball sampling)***, select one of the following:**
Intermediary provides information to potential participants who then contact the researchers

3.0 **If contact will be made through an intermediary, explain why the intermediary is appropriate and describe what steps will be taken to ensure participation is voluntary:**
Patients with scoliosis will be informed of the study and invited to participate by the clinic coordinator (not a study team member) when arranging clinic visits. The information will be sent by email or discussed by phone when booking the clinic visit. The booking is not dependent on patient's agreement to participate. Once informed of the study by the coordinator and the information letter, patients will be invited to contact the researcher to learn more about the project and arrange participation in the study. The coordinator will explicitly mention that patient may refuse her participation in the study. Patients will also be given the option to leave their contact information to the clinic coordinator with an implied authorization for the researchers contact them to explain the study in details after attending the clinic. This will afford time for the patients and their family to reflects on the large amount of information routinely provided at the clinic and allow researchers to contact willing subjects at a time of their choice.

Only persons who would otherwise have access to the patients' medical records for clinical care will be screening those same records for research eligibility (Nurse practioner / Clinical engineer/ clinic coordinator depending on availability). Study staff not involved in Scoliosis clinic clinical care will not be screening medical records to determine study eligibility, unless the potential participants have previously consented to this process (None have at this point but the scoliosis clinic team is in discussion to determine whether we should routinely ask about their willing to do so in the future, after seeking advice from the ethics board).

4.0 **Provide the locations where participants will be recruited,** *(i.e. educational institutions, facilities in Alberta Health Services or Covenant Health, etc)***:**
The coordinator works at the University of Alberta Hospital, as a nurse. She will be contacting the patient at their home as she books their clinic visit. Contact may be by phone, email or mail as these are the usual modes of how booking is done for the clinic.

ID: Pro00011552 View: 4.4 Informed Consent Determination
Status: Approved

4.4 Informed Consent Determination

1.0 * **Describe who will provide informed consent for this study (select all that apply):**
Not all participants will be competent to give informed consent
Third party consent will be sought

2.0 **How is consent to be indicated and documented? Select all that apply:**
Signed consent form

3.0 **What assistance will be provided to participants, or those consenting on their behalf, who have special needs** *(eg non-English speakers, visually impaired, etc)***:**
Researchers will be available to answer question on site when the consent/assent form is signed. Forms will be emailed ahead of participating to ensure the participants have plenty of time to digest the information. The researchers could be contacted via phone or email to answer questions ahead of participation if needed. Those under 18yo will be invited to provide assent and parental consent will be obtained.

4.0 **If at any time <u>a participant wishes to withdraw, end, or modify their participation in the research or certain aspects of the research</u>, describe the procedures and the last point at which it can be done:**
Participants may do so at anytime.

5.0 **Describe the circumstances and limitations of <u>data withdrawal</u> from the study, including the last point at which it can be done:**
Data withdrawal is possible by patient request at anytime before the analyses are completed.

6.0 **Will this study involve any group(s) where non-participants are present? For example, classroom research might involve groups which include participants and non-participants.**
○ Yes ● **No**

7.0 **Describe the incentives and/or reimbursements, if any, to participants and provide justification:**
Patients will be asked to commit time for the assessments, the treatments (individual and weekly group classes), and the home exercise routine. No funds are available to compensate patients for their time. Evaluations and treatments are provided at no charge to patients or their insurance. Transportation to the evaluations and treatments are the responsibility of the patients/ parents and will not be reimbursed. Participants will be provided with parking coupons for each visit to Corbett Hall.

ID: Pro00011552 View: 4.6 Authorized Representative or Third Party Consent
Status: Approved

4.6 Authorized Representative or Third Party Consent

1.0 * **Explain why the participant is unable to give informed consent** *(eg, young age, mental, or physical condition, etc)***:**
Participant will be considered as unable to give informed consent because all enrolled are expected to be younger than 18 years. Since enrolling in the study will require parental support (driving to treatments for up to 6 months, assent from patients and parental consent are the ideal informed consent strategy for this study.

Will the participant who is not competent to give full informed consent be asked to give assent?
(•) **Yes** ( ) No

2.0 **Provide details. If applicable, attach a copy of assent form(s) in Section 7: Documentation section:**
Assent forms will be provided by email (of fax as needed) prior to the visit and signed during the visit. Opportunities to ask question by phone or before beginning study procedures will be available.

In cases where participants gain capacity to consent during the study, how will they be asked to provide consent on their own behalf?
3.0 We do not anticipate that many patients will be followed-up until 18 years old when they would be able to consent on their own. If they do, we will have them complete a consent form once they reach the age of consent. Nevertheless, all subjects will complete an assent form to ensure that they are informed of the nature of what is expected of them in the study.

ID: Pro00011552 View: 4.8 Study Population Categories
Status: Approved

4.8 Study Population Categories

1.0 * **This study is designed to TARGET or specifically include the following** *(does not apply to co-incidental or random inclusion)*. **Select all that apply:**
Women
Children/Youth

ID: Pro00011552 View: 5.1 Research Methods and Procedures
Status: Approved

5.1 Research Methods and Procedures

Some research methods prompt specific ethic issues. The methods listed below have additional questions associated with them in this application. If your research does not involve any of the methods listed below, ensure that your proposed research is adequately described in Section 2.0: Study Objectives and Design or attach documents in Section 7.0 if necessary.

1.0 * **This study will involve the following** *(select all that apply)*
The list only includes categories that trigger additional page(s) for an online application. For any other methods or procedures, please indicate and describe in your research proposal in the Study Summary, or provide in an attachment:
Surveys and Questionnaires (including internet surveys)
Sound or image data involving participants (other than audio or video-recorded interviews or focus groups)
Health and Biological Specimen Collection
Registries and Databases (including Biobanks)
Radiation: Any test or procedure that may involve exposure to radiation (including screening chest x-ray)

2.0 **Is this study a Clinical trial?** *A clinical trial is any research study that prospectively assigns human participants or groups of humans to one or more* ***health-related intervention(s)*** *to evaluate the effects on* ***health outcomes;*** *does not include randomized controlled trials � RCT � outside of clinical settings)?*
( ) Yes (•) **No**

3.0 **For registered clinical trial(s), provide registry and registration number, if available:**
not registered yet

4.0 **Internet-based research**

4.1 Will you be doing any internet-based research that involves interaction with participants?
( ) Yes (•) **No**

4.2 If YES, will these interactions occur in private spaces (eg. members only chat rooms, social networking sites, email discussions, etc)?
( ) Yes (•) **No**

4.3 Will these interactions occur in public space(s) where you will post questions initiating and/or maintaining interaction with participants?
( ) Yes (•) **No**

5.0 **If you are using any tests in this study diagnostically, indicate the member(s) of the study team who will administer the measures/instruments:**

Test Name	Test Administrator	Organization	Administrator's Qualification
	There are no items to display		

6.0 **If any test results could be interpreted diagnostically, how will these be reported back to the participants?**
- The blood tests are experimental and may suggest that subject with different values on the test have (they should all be based on selection criteria) or will develop scoliosis, or will see their scoliosis curve progress in the future. Results could be shared with the patients in a letter sent by the PI with a note specifying that these are experimental results that may suggest that monitoring by a physician is needed.

ID: Pro00011552 View: 5.6 Sound or Image (other than audio- or video-recorded interviews) or Material Created by Participants
Status: Approved

5.6 Sound or Image (other than audio- or video-recorded interviews) or Material Created by Participants

Explain if consent obtained at the beginning of the study will be sufficient, or if it will be necessary to obtain consent at different times, for different stages of the study, or for different types of data:
1.0 We will not require consent at different times. For patient having provided assent at baseline but reaching age of consent and judged

competent to provide consent by the evaluator, the evaluator will provide an information letter and consent form to complete during follow-up visits.

If you or your participant�s audio- or video-records, photographs, or other materials artistically represent participants or others, what steps will you take to protect the dignity of those that may be represented or identified?

2.0 The head will be cropped off for the analyses of the scans to maintain privacy of the participants. If a participant's scans is to be used in presentations and publications, the standard hospital release form will be completed by the patients prior to presentation or publication.

Who will have access to this data? For example, in cases where you will be sharing sounds, images, or materials for verification or feedback, what steps will you take to protect the dignity of those who may be represented or identified?

3.0 A research assistant will crop the head of the scans. A PhD student in Rehabilitation Medicine will complete the scan analyses in private offices with identifying features of the head removed and anonymous scan id. Breasts of females will be hidden by stick-on bras prior to scanning to maximize privacy. Reporting of the measures is only via measurements not the scan themselves.

4.0 **When publicly reporting data or disseminating results of your study (eg presentation, reports, articles, books, curriculum material, performances, etc) that include the sounds, images, or materials created by participants you have collected, what steps will you take to protect the dignity of those who may be represented or identified?**

We will ask for a volunteer completion of a release form. Head features will be removed from scans used and no personal identification will be used. Breasts of females will be covered by stick-on bras.

5.0 **What opportunities are provided to participants to choose to be identified as the author/creator of the materials created in situations where it makes sense to do so?**

Does not Apply

6.0 **If necessary, what arrangements will you make to return original materials to participants?**

Does not apply

ID: Pro00011552 View: 5.7 Interviews, Focus Groups, Surveys and Questionnaires
Status: Approved

5.7 Interviews, Focus Groups, Surveys and Questionnaires

1.0 **Are any of the questions potentially of a sensitive nature?**

○ Yes ● **No**

If YES, provide details:

The questionnaires are normally completed as part of routine visits to the scoliosis clinic. Questions are part of five domains of scoliosis related quality of life (function, pain, mental health, self-image and satisfaction).

The scanning procedure, since the torso is disrobed, could be perceived as sensitive by some patients and we have explained our effort to make them feel comfortable during the scans and to maintaining dignity and privacy.

2.0 **If any data were released, could it reasonably place participants at risk of criminal or civil law suits?**

○ Yes ● **No**

If YES, provide the justification for including such information in the study:

3.0 **Will you be using audio/video recording equipment and/or other capture of sound or images for the study?**

● **Yes** ○ No

If YES, provide details:

The scanner generates a regular picture of the torso of the patient and a 3D shape of the torso.

ID: Pro00011552 View: 5.11 Health and Biological Specimen Collection
Status: Approved

5.11 Health and Biological Specimen Collection

1.0 * **Indicate health or biological specimen(s) that will be collected** *(for example, body tissues or fluids, be specific)*:

Blood sample (10 ml.)

2.0 * **This study will involve the following** *(select all that apply)*:

Collection of sample for immediate use

Other

If OTHER, provide details:

The immediate use is defined here as sample extracted by the laboratory, packaged and sent to Centre hospitalier Ste-Justine to Dr Alain Moreau's lab for the analyses described in the project protocol.

3.0 **Explain how the specimen will be collected:**

A 10-ml blood sample will be taken at the Campus plaza by a research nurse employed by the Canadian blood service repository by the means of routine clinical standards, and sent to Montr�al for analysis.

4.0 **Explain how the specimen will be stored, and for how long:**

Specimen will only be stored frozen in dry ice shortly until shipped and analyzed in Montr�al.

5.0 **Specify all intended uses of collected specimen:**

Collected specimens are intended only for use in this study.

ID: Pro00011552 View: 5.12 Registries and Databases (including Biobanks)
Status: Approved

5.12 Registries and Databases (including Biobanks)

1.0 * **Where will the databases be located? Specify if the database will be under Canadian or foreign jurisdiction.** Note that data housed on US servers fall under the US Patriot Act. At a minimum, participants should be informed of this potential breach in confidentiality.

Databases will be located in a password protected computers in Dr. Parent's lab (Corbett Hall).

Radiographs and SRS-22 questionnaires data will be extracted from the Edmonton Scoliosis Database stored on a password protected computer of the Edmonton Scoliosis clinic and then Stored in Dr. Parent's lab.

Surface topography scans and extracted measures will be stored in the local acquisition computers in the Clinical science building Scoliosis surface topography lab.

All computer are password protected and database files are also password protected.

2.0 * **Who will have access to the databases? How is that access determined?**
Study team personnel only using user name and password protection of access.

3.0 **Biobanks: Where will the biobank(s) be located? Specify if the biobank(s) will be located in Canadian or foreign jurisdiction.**
Canada

4.0 **Provide information if material is linked or de-linked:**
Material will be de-linked as soon as possible.
We will query the clinic database using personal information but will store the extracted data with the rest of the study data collected using study id's.

ID: Pro00011552 View: 5.13 Biohazard Safety
Status: Approved

5.13 Biohazard Safety

1.0 **AMENDMENT OR RENEWAL**: If this application is for the amendment or renewal of a pre-existing clinical study: have new biohazards and/or manipulations been added to the research that were not identified in the original study protocol?
*
Not Applicable (if this application is for a new study or a pre-existing non-clinical study)

If you selected **NO**, this amendment or renewal is exempt from requiring further review by the EHS Biosafety Division and the *original biohazard approval remains valid*. You do not need to respond to any of the questions below.

If you selected **YES**, this amendment or renewal is considered new research - please respond to question 2.0 below.

2.0 **Will your research involve the use of one or more of the following? Provide a response for each item.**

Answer	Description
Yes **No** (selected)	Risk group 2, 3 or 4 viruses, bacteria, fungi, parasites or eukaryotic cell lines
Yes **No** (selected)	Environmental specimens suspected to contain risk group 2, 3 or 4 microbes
Yes **No** (selected)	Large-scale single volume culture in excess of 10 litres for any microbe or eukaryotic cell line
Yes **No** (selected)	Microbial toxins
**Yes** (selected) No	Human clinical specimens, including blood or other body fluids, or primary culture of human cells
Yes **No** (selected)	Xenotransplant studies involving vertebrate donors and/or recipients
Yes **No** (selected)	Genetic therapy studies involving vertebrate donors and/or recipients
Yes **No** (selected)	Genetic manipulation involving virulence genes from risk group 2, 3 or 4 microbes, mammalian oncogenes, mammalian cytokine or interleukin genes, or microcide resistance genes

If you answered YES to any of the above, you will need to apply for Biohazards approval. Send a copy of your grant application or experimental plan detailing the planned use of these biohazards to:

Biosafety Division
Environmental Health and Safety
EHS_RSR@ehs.ualberta.ca

In your correspondence, be sure to include the Principal Investigator�s name and department, the project title, and the name of the funding source, if applicable

ID: Pro00011552 View: 5.14 Radiation Safety
Status: Approved

5.14 Radiation Safety

1.0 **Will your research involve:**
There are no items to display

"If you take part in this research, you will be exposed to a very small amount of radiation. The risk from this amount of radiation has been categorized by the Radiation Safety Committee as 'very low'."

Note: If you have checked either of these boxes, a separate application to the Radiation Safety Committee (RSC) for approval is not usually required.

2.0 **Will your research involve exposure of subjects aged 0-17 years to any amount of ionizing radiation?**
Yes (selected) No

Regardless of how little radiation is involved, this requires separate application to the Radiation Safety Committee (RSC) for approval. Please complete section 3.0 and contact the committee as indicated below.

3.0 **Will your research involve any of the following at screening, baseline or follow-up?** *(Check all that apply)*
X-rays of the skull, facial bones, neck, spine, thorax, abdomen, pelvis or hip

Note: if you checked any box in section 3.0, you need to apply for RSC approval.
To apply for RSC approval, e-mail a copy of the complete research protocol, including patient information sheet, to: radnsfty@ualberta.ca

In most cases, RSC approval will be issued in 1-2 days, unless otherwise notified. Some rewording of the patient information sheet is often required. Protocol amendment is rarely necessary.

For further information, contact the RSC at:

Dr. R. Lambert
Chair, Alberta Health Services/University of Alberta Regional Radiation Safety Committee
2A2.18 WMC, UAH Site
Ph. 407-8223, Fax 407-3853,
E-mail: radnsfty@ualberta.ca

ID: Pro00011552 View: 6.1 Data Collection
Status: Approved

6.1 Data Collection

1.0 * **Will the researcher or study team be able to identify any of the participants at any stage of the study?**
(•) **Yes** ( ) No

Primary/raw data collected will be *(check all that apply)***:**
2.0 Indirectly identifying information-the information can reasonably be expected to identify an individual through a combination of indirect identifers (eg date of birth, place of residence, photo or unique personal characteristics, etc)

If identifying information will be removed at some point, when and how will this be done?
3.0 During the patient visit. We will assign a code which will appear in all data collection forms and in the images saved and on the blood sample extracted. The code will be used to match the different pieces of data to the same patient. We will keep coded identifying information in a separate file for the PI in order to be able to contact patients who would potentially need a referral.

If this study involves secondary use of data, list all original sources:
4.0 Data from the Edmonton Scoliosis database will be extracted as described in the informed consent procedures with the permission of the patient: radiograph measures at baseline, 3, 6, 12 months follow-up, SRS-22 questionnaires at the same times.

In research where total anonymity and confidentiality is sought but cannot be guaranteed *(eg. where participants talk in a group)* **how will confidentiality be achieved?**
5.0 Total anonymity is not sought per se. Participants will exercise in groups and get to meet other participants. Participants will be informed of the need to maintain confidentiality of personal information learned in the study. They will also be asked to refrain from informing the evaluator of their study group or of that of other participants.

ID: Pro00011552 View: 6.2 Data Identifiers
Status: Approved

6.2 Data Identifiers

1.0 * **Personal Identifiers:** will you be collecting any of the following *(check all that apply)***:**
Full Name
Address
Full Postal Code
Telephone Number
Email Address
Age at time of data collection

If OTHER, please describe:

2.0 **Will you be collecting any of the following** *(check all that apply)***:**
There are no items to display

If OTHER, please describe:

3.0 **If you are collecting any of the above, provide a comprehensive rationale to explain why it is necessary to collect this information:**
The contact information is necessary to be able to contact the patients if when analyzing the data we find reasons to refer the patients for medical care or investigation.
Personal information will also be used to query the clinic database and retrieve radiograph information and questionnaire information and link it with the rest of the study data. This linkage allows avoiding having to request that additional radiographs or questionnaires be completed.

4.0 **Specify what <u>identifiable</u> information will be RETAINED once data collection is complete, and explain why retention is necessary. Include the retention of master lists that link participant identifiers with de-identified data:**
All the information above will be retained but de-linked from the study data until all results have been analyzed and published in case findings suggest a referral of the patient is needed. After that time we will destroy the file containing the personal information.

5.0 **If applicable, describe your plans to link the data in this study with data associated with other studies (e.g within a data repository) or with data belongong to another organization:**
Personal identifiers above will be used to query the data from the Edmonton scoliosis database.
Once extracted and copied to our study database, the date will be de-linked.

ID: Pro00011552 View: 6.3 Data Confidentiality and Privacy
Status: Approved

6.3 Data Confidentiality and Privacy

1.0 * **How will confidentiality of the data be maintained? describe how the identity of participants will be protected both during and after research.**
Study document in locked filing cabinet, computer files on a password protected computer, blood samples shipped with only the study id code and not personal information.

2.0 **What privacy education/training do members of the team have prior to their access to data? How will those who have access to the data be made aware of their responsibilities concerning privacy and confidentiality?**
Basic CITI course in the Protection of Human Research Subject Nov. 2007 Paul Braunschweiger Ph.D., U. of Miami, online CITI Course Coordinator (Eric Parent)

FGSR online ethics course, section on ethics of REHAB 600 and 601 for Elise Watkins and Sanja Bosnjak.

Doug Hill is a member of the HREB panel B

If you involve colleagues, assistants, transcribers, interpreters and/or other personnel to carryout specific research tasks in

3.0 **your study, how will you ensure that they properly understand and adhere to the University of Alberta standards of data privacy and confidentiality?**
Should the need arise for such an involvement, I will suggest that they complete the Tri-council training and quizzes online as linked from the HERO link section.

Data Access

*** 4.1 Will identifiable data be transferred or made available to persons or agencies outside of the research team?**
Yes No

4.0 **4.2 If YES, describe in detail what identifiable information will be released, to whom, why they need access, and under what conditions? What safeguards will be used to protect the identity of subjects and the privacy of their data.**

4.3 Provide details if identifiable data will be leaving the institution, province, or country *(eg. member of research team is located in another institution or country, etc.)*
Only one member is located in another institution (Alain Moreau, Hopital Ste-Justine, Montr�al). Blood samples sent to Alain will be coded with the study ID and no personal information will be sent outside of the institution.

ID: Pro00011552 View: 6.4 Data Storage, Retention, and Disposal
Status: Approved

6.4 Data Storage, Retention, and Disposal

Describe how research data will be stored, e.g. digital files, hard copies, audio recordings, other? Specify the physical location and how it will be secured to protect confidentiality and privacy. (For example, study documents must be kept in a locked filing cabinet and computer files are encrypted, etc.)
1.0 On computer and in locked file cabinet in room 3-78 of Corbett Hall. (Dr Parent's lab).
Blood samples will be collected at the University of Alberta Hospital Lab and be shipped to the laboratory of Dr Alain Moreau at Hospital Ste-Justine in Montr�al and results will be returned to Dr. Parent's lab). Blood samples will be destroyed in Montr�al after analysis.

2.0 **If you plan to destroy your data, describe when and how this will be done. Indicate your plans for the destruction of the identifiers at the earliest opportunity consistent with the conduct of the research and/or clinical needs:**
Once analyses are completed and results published we will destroy the personal identifier files.

3.0 **You must keep your data for a minimum of 5 years according to GFC Policy 96.2. How will you provide for data security during this time?**
Data will be in password protected files and in locked cabinets in Dr. Parent's lab.

ID: Pro00011552 View: 7.1 Documentation
Status: Approved

7.1 Documentation

Add documents in this section according to the headers. Use Item 12.0 "Other Documents" for any material not specifically mentioned below.

Sample templates are available in the HERO Home Page in the **Forms and Templates**, or by clicking HERE.

Important: Please do not use .docx files as attachments. It is recommended you convert these files first to .doc (standard Word document files) before attaching.

1.0 **Recruitment Materials:**

Document Name	Version	Date	Description
There are no items to display			

2.0 **Letter of Initial Contact:**

Document Name	Version	Date	Description
letter of initial contact revised | History	0.01	9/14/2010 10:59 PM	
Letter of initial contact | History	0.01	8/6/2010 1:54 PM	

3.0 **Informed Consent / Information Document(s):**

3.1 What is the reading level of the Informed Consent Form(s):
Assent form reading ease is 6.7; Parental consent form is 7.7: for Information Sheet is 10.0.

3.2 Informed Consent Form(s)/Information Document(s):

Document Name	Version	Date	Description
Information letter revised (AMENDED) | History	0.03	3/21/2011 2:26 PM	
Parental consent revised | History	0.01	9/14/2010 11:01 PM	
Information letter | History	0.02	8/12/2010 10:52 AM	
Parental consent forms | History	0.01	8/6/2010 1:54 PM	

4.0 **Assent Forms:**

Document Name	Version	Date	Description
Assent revised (AMENDED) | History	0.03	3/21/2011 2:27 PM	
Assent form | History	0.02	8/12/2010 10:48 AM	

5.0 **Questionnaires, Cover Letters, Surveys, Tests, Interview Scripts, etc.:**

Document Name	Version	Date	Description
There are no items to display			

6.0 **Protocol:**

Document Name	Version	Date	Description
CIHR Grant Application Draft | History	0.02	8/12/2010 10:45 AM	
Glenrose Rehabilitation Hospital Research Grant | History	0.01	8/4/2010 2:12 PM	
SRS Small Exploratory Grant Application | History	0.01	8/4/2010 2:09 PM	

Investigator Brochures/Product Monographs *(Clinical Applications only)***:**

	Document Name	Version	Date	Description
7.0		There are no items to display		

Health Canada No Objection Letter *(NOL)***:**

	Document Name	Version	Date	Description
8.0		There are no items to display		

Confidentiality Agreement:

	Document Name	Version	Date	Description
9.0	Non-disclosure Agreement | History	0.01	8/4/2010 1:43 PM	

Conflict of Interest:

	Document Name	Version	Date	Description
10.0		There are no items to display		

Other Documents:

For example, Study Budget, Course Outline, or other documents not mentioned above

	Document Name	Version	Date	Description
11.0	response to requested changes | History	0.01	9/14/2010 11:05 PM	

ID: Pro00011552 View: SF - Final Page

Status: Approved

Final Page

You have completed your ethics application! Please select "Exit" to go to your study workspace.

This action will NOT SUBMIT the application for review.

Only the Study Investigator can submit an application to the REB by selecting the "SUBMIT STUDY" button in My Activities for this Study ID:Pro00011552.

You may track the ongoing status of this application via the study workspace.

Please contact the REB Administrator with any questions or concerns.
